# High Performance Valve Seat Materials for CNG Powered Combustion Engines

**DOI:** 10.3390/ma14174860

**Published:** 2021-08-26

**Authors:** Andrzej Romański, Elżbieta Cygan-Bączek

**Affiliations:** 1 Faculty of Metals Engineering and Industrial Computer Science, AGH University of Science and Technology, 30 Mickiewicz Avenue, 30-059 Krakow, Poland; 2 Center of Advanced Manufacturing Technology, Łukasiewicz Research Network—Krakow Institute of Technology, Zakopiańska 73 Str., 30-418 Krakow, Poland; elzbieta.baczek@kit.lukasiewicz.gov.pl

**Keywords:** valve seats, CNG engine, resistance to wear, high speed steel, infiltration

## Abstract

The conventional copper infiltrated high speed steel (HSS) valve seats used in gasoline engines are not suitable for CNG combustion because the exhaust gas temperature is at least 80 °C higher, which drastically shortens the service life of the engine valves. Therefore, a proprietary high-alloy HSS-base material was designed to combat hot corrosion and mechanical wear of valve seat faces in CNG fuelled engines. A batch of −100 mesh water atomized HSS powder was commissioned. The powder was vacuum annealed in order to reduce oxygen content and increase its compressibility. To improve the final part machinability, 1.2% MnS was admixed to the HSS powder prior to compaction. The green compacts were sintered at 1135 °C in nitrogen to around 83% TD and subsequently infiltrated with a copper alloy. After installing the valve seat components on a cylinder head, the engine was tested for 100 h according to the automotive industry valve seat wear test procedures. Both the periodic 8-h checks as well as the final examination of the valve seats showed very slow wear, indicating their suitability for CNG powered engines.

## 1. Introduction

Natural gas vehicles (NGVs) are defined by Natural Gas Vehicles Global (formerly the International Association for Natural Gas Vehicles) as all land-based motor vehicles, from two wheelers through to off-road [1]. Nowadays, as a results of introducing the new WLTP homologation process and the need of reducing pollution and decarbonisation across the transport sector, a strong interest in using compressed natural gas (CNG) or liquefied natural gas (LNG) as a fuel for powering vehicles is observed, especially in public transport, trucks and passenger cars. According to the Vehicle Catalogue 2019, there are 68 gas vehicles currently available in Europe, from passenger cars and light commercial vehicles to heavy-duty trucks and buses. The number of registrations of new CNG powered cars recorded in the EU in 2019 approached 83,000. The top five countries were Italy (with 55.2% of total passengers cars registrations), Germany (10.9%), Spain (7.8%), Sweden (7.6%) and Belgium (5.0%) [2]. The NGV fleet in Europe has about 1.5 million vehicles, while the rest of the world has more than 25 million [3]. The constantly growing demand for natural gas fuelled vehicles has forced the automotive industry to modify engines in order to meet high performance criteria for gasoline or natural gas.

Valve seat inserts are used for intake and exhaust valves in internal combustion piston-engines. They are exposed to severe working conditions in an aggressive environment, including wear by erosion and corrosion, thermal fatigue and oxidation at high temperatures. For both gasoline and CNG, the exhaust gas temperature depends on the engine speed and loading conditions. According to Ref [4], for the same working conditions the exhaust gas temperature is almost 100 °C higher for CNG as compared to gasoline, and can exceed 900 °C. This is due to the slower combustion of CNG, which continues to burns after the exhaust valve has been opened, which releases a lot of heat into the exhaust system. During the whole engine life, the valve materials are exposed to continuous, destructive temperature fluctuations. Hence, their resistance to wear and thermal fatigue is crucial in the prevention of valve failure when the engine runs at a high speed for a long time [4].

As reported in Ref [5], coated valve seat inserts are often used in order to improve the bonding of the inserts to the cast Al alloy cylinder head as well as to increase their heat conductivity and resistance to wear and oxidation. A wide variety of coating compositions are used, including Cu, Sn, Zn, Ag, Cu-Zn, Al, Al-Si or Si. This technique has a disadvantage because the coating deposition by electroplating, hot dipping, physical or chemical methods (PVD, CVD) or flame spraying may markedly contribute to higher production costs. In Ref [6] it is proposed to use Stellite-12 coated seat valves to secure wear resistance in a dry environment characteristic of gas fuel. Moreover, a change in valve and valve seat angle from 45° to 30° has been recommended in Refs [6,7] in order to increase the valve seat resting area.

The powder metallurgy (PM) technique is the method of choice for the production of valve seat inserts. The PM process allows the manufacture of parts and metallic components at low costs, great flexibility in alloy composition and microstructural control. HSS-based materials have proven to be particularly well suited for valve seat inserts. However, HSSs alone cannot be used for valve seat inserts due to a high risk of overheating the engine head, leading to cracks associated with the stresses generated by the thermal expansion mismatch between the valve seat insert and the engine head. Apparently, an application of copper infiltrated HSS valve seat components is the simplest way to avoid cracking and to increase the overall heat conductivity. To date, valve seats have been mainly manufactured from copper infiltrated M3/2 grade HSS containing Cu3P, pure iron and/or niobium carbide additions [8,9,10,11]. These materials were examined for microstructure and the effect of heat treatment on properties. Although much is known about the valve seat behaviour in gasoline engines, fundamental research on using such materials in CNG fuelled engines is still lacking.

The aim of this study was to design and test a new valve seat material based on a proprietary high-alloy, AGH60 HSS, infiltrated with a copper-nickel alloy for application in CNG powered four-stroke piston-engines. Taking into account higher temperatures of exhaust gases when the CNG is used as a fuel, the AGH60 HSS was designed to attain better application and higher temperature properties compared to the M3/2 HSS grade. The microstructural investigations were supplemented with data acquired from a 2-cylinder engine performance test.

## 2. Experimental Procedure

To meet high temperature resistance to wear of valve seat, a new HSS powder was developed to ensure sufficient amount of carbides in the microstructure between 900 and 1200 °C, mainly MC and M_6_C type, which guarantee a high performance of the steel at elevated temperatures. The thermodynamic calculations were done by Thermo-Calc Software. The newly developed HSS powder was designated as AGH60, and its chemical composition is given in Table 1.

**Figure 1:**
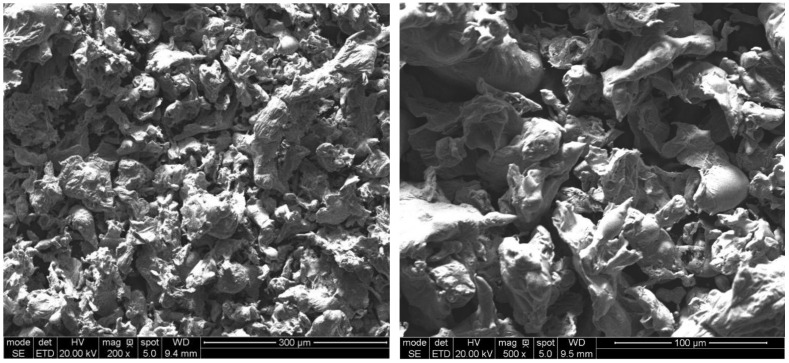
AGH60 water atomised powder.

In order to achieve good compacting properties, the AGH60 powder was produced by water atomisation and screened to −100 mesh (<150 µm). The particle shape of the experimental powder is given in Figure 1.

To reduce the oxygen content and improve compressibility, the powder was vacuum annealed at 935 °C for 30 min and cooled at 3 K/min to room temperature. The valve seat rings were prepared by the conventional press and sinter route. The porous skeletons were subsequently infiltrated with a copper-nickel infiltrant. Prior to compaction, 0.5 wt.% LicowaxC lubricant (provided by Clariant AG, Muttenz, Switzerland) and 1.2 wt.% MnS (provided by New Materials Development, Rosenheim, Germany) were admixed with the HSS powder for better powder compressibility (LicowaxC) as well as to improve part machinability and to decrease friction at the valve seat ring/valve head interface (MnS).

All powders were mixed in a Turbula-type mixer for half an hour. Thick wall tubes with an inner diameter of 22 mm, an outer diameter of 30 mm and a height of 8 mm were cold compacted on a double-action press in a carbide-lined die under a pressure of 600 MPa. The green rings were sintered in a laboratory tube furnace in nitrogen. Two 20 min holds, at 550 and 950 °C, were incorporated into the heating cycle in order to facilitate lubricant removal and to homogenise the temperature inside the sintered part, respectively. After an hour hold at 1135 °C, the rings were furnace cooled to 850 °C and then transferred to a water-jacketed cooler.

**Figure 2:**
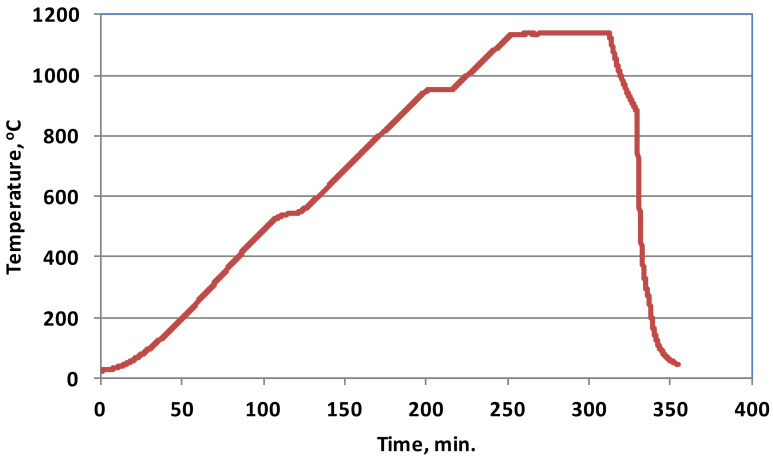
Temperature profile of the sintering cycle.

The sintering cycle is presented in Figure 2.

The sintered skeletons were contact infiltrated with Cu-5 wt.%Ni infiltrant at 1145 °C for 10 min in vacuum. The infiltrated parts were furnace cooled down to 325 °C.

**Figure 3:**
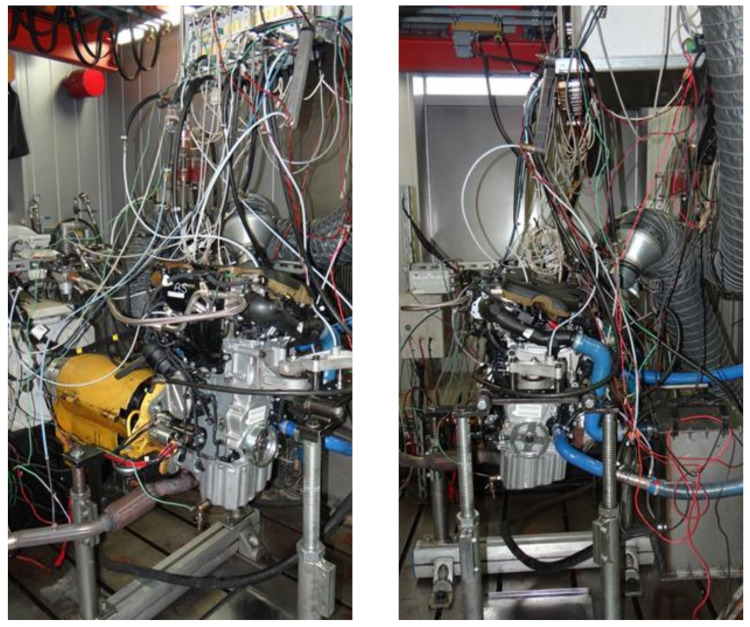
The engine on a dynamometer.

**Figure 4:**
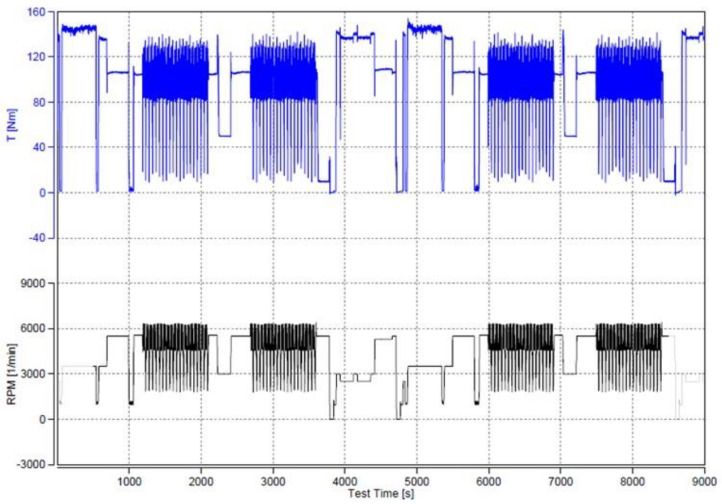
The engine loading condition.

**Figure 5:**
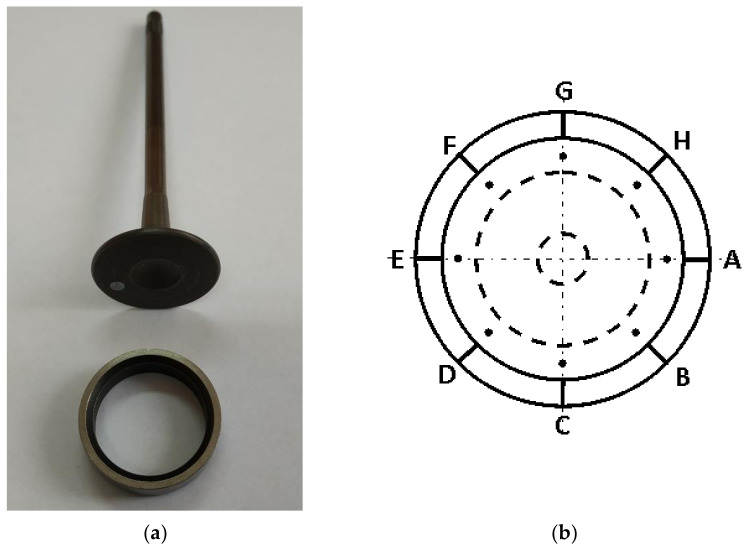
A pair of valve stem and seat insert (**a**); measurement points marked with black dots (**b**).

The infiltrated specimens were CNC machined into the required valve seat ring shape and size (see Figure 5) and installed in a cylinder head of a four-stroke TwinAir 0.9 SGE TC CNG 80HP CNG powered engine. The engine was then subjected to 100 h accelerated test according to the automotive industry valve seat wear test procedures. The testing cycle was divided into two 50 h checks. Moreover, every 8 h, the engine was visually checked for oil and coolant leaks, loose fasteners, cracks in brackets, etc. As shown in Figure 3, the engine was installed on a dynamometer and tested on brake, at a very high engine speed and full load. After the test, the wear depth was measured on each seat and seat valve. Typical loading conditions are illustrated in Figure 4.

After the 100 h test, the faying face of each valve seat was examined in eight places for depth of wear, as indicated schematically in Figure 5.

## 3. Results and Discussion

The as-sintered densities of porous skeletons were calculated from their mass and dimensions, whereas densities of the infiltrated samples were measured using the Archimedes’ principle. In order to calculate the porosity of the investigated materials, the theoretical density was estimated on the basis of the rule of mixtures. The infiltrated specimens were also tested for Vickers hardness on polished cross-sections at a load of 1 kG. The results are summarised in Table 2

The porosity of sintered skeletons is around 35% and seems suitable for the purpose. The seat valve inserts installed in the head of an engine are subjected to high temperatures during combustion cycles. In order to improve the heat transfer, it was decided to maintain elevated porosity and fill the pores with copper alloy, which conducts heat much better than the AGH60 HSS. After infiltration, the material had only closed porosity.

**Figure 6:**
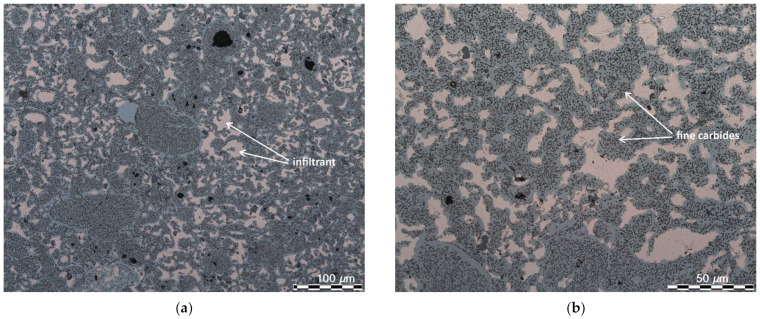
As-infiltrated microstructure of a valve seat: (**a**) 200×, (**b**) 500×, LM.

The infiltrated specimens were subjected to microstructural observations. Selected micrographs are presented in Figure 6.

As it can be seen, all pores are almost completely filled with copper alloy. There are also some isolated pores which were not penetrated by the liquid infiltrant. Presumably, most of them were closed during sintering, but total porosity is less than 1%. The microstructure of the infiltrated specimens consists of uniformly distributed carbides. According to the calculations performed using Thermo-Calc Software, a low cooling rate leads to a microstructure which consists of three types of carbides, e.g., MC, M6C and M23C6. The total contribution of the carbides at room temperature is nearly 16 vol. %. Moreover, the newly developed high speed steel is characterised by increased content of carbides at temperatures similar to those which the valve seats of CNG powered engines are subjected to. As presented in Table 3, the total content of carbides in ASP60 HSS at temperatures between 900 and 1200 °C is markedly higher when compared to the commonly used M3/2 HSS grade.

The depth of seat wear was measured according to the valve and valve seat wear test procedure used in the automotive industry. The obtained results are summarised in Table 4.

The data presented in Table 3 indicates that valve seats no. 1 and 2, mounted in cylinder no. 1, were worn slower then valve seats no. 3 and 4, mounted in cylinder no. 2. This is due to the lower temperature of the engine head near cylinder no. 1, which is due to more intense cooling. Despite differences in wear measurements between the valve seats, the overall wear rate is lower than expected. The 100 h test performed on an engine installed on a dynamometer corresponds to approximately 150,000 km of engine mileage. Therefore, the recorded data should be considered as valve seat insert positioning rather than their abrasion.

**Figure 7:**
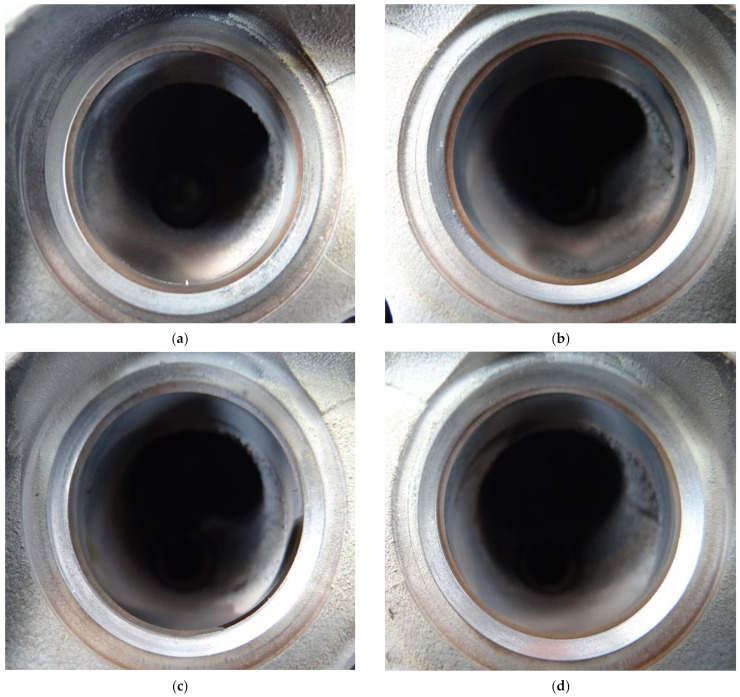
Valve seat inserts after completion of 100 h test. Valve seat no. 1 (**a**), 2 (**b**), 3 (**c**) and 4 (**d**).

The valve seat inserts were also inspected visually with a laparoscope for any damage to the seat sides—Figure 7.

As it is evident from Figure 6, there are no visible signs of plastic deformation due to insufficient strength of the investigated material at elevated temperatures. The seat sides retain shape and look smooth, which also proves the suitability of AGH60 HSS infiltrated with copper-base alloy for manufacturing valve seat inserts for CNG fuelled combustion engines.

## 4. Concluding Remarks

The presented research programme is of high importance, especially when valve seats wear test results are considered. It has been proved that:

1. the infiltration of porous skeletons made of sintered AGH60 HSS is a suitable manufacturing process for production of valve seat inserts intended for CNG fuelled engines;
2. both the periodic 8-h checks and the final examination of the valve seats showed very slow wear, indicating their suitability for CNG powered engines.

A commercial application of this material should be followed by further detailed examinations, depending on testing procedures for a given type of engine. Nevertheless, the obtained results are very promising and show that the newly developed material can withstand the demanding working conditions of CNG fuelled engines.

## Figures and Tables

**Table 1:** Chemical composition of AGH60 powder (wt.%).

	C	Cr	W	Mo	V	Co	Fe
AGH60	2.22	3.80	6.15	6.45	5.38	9.22	bal

**Table 2:** Densities, porosities and Vickers hardness numbers of investigated specimens.

Sample ID	After Sintering		After Infiltration		
	Density, g/cm^3^	Porosity, %	Density, g/cm^3^	Porosity, %	HV1
S1	5.14	36.5	8.02	3.9	367 ± 21 *
S2	5.20	35.8	7.92	3.8	377 ± 36 *
S3	5.31	34.4	8.07	3.5	379 ± 32 *
S4	5.26	35.1	8.04	3.7	373 ± 27 *
average	5.23	35.5	8.01	3.7	374 ± 29

*—mean value from 10 measurements; confidence intervals were estimated at 90% confidence level.

**Table 3:** Microstructure composition of M3/2 and AGH60 HSS calculated by Thermo-Calc Software.

Temperature, °C	M3/2			AGH60				
	Austenite	MC-Type	M6C-Type	Austenite	MC-Type	M6C-Type	M7C3-Type	Other
room temp.	77.3%	2.8%	1.1%	71.1%	4.0%	4.7%	-	7.2% (M23C6), 12.7% (FCC), 0.3% (Laves phase)
900	83.3%	7.0%	9.7%	73.6%	15.1%	9.3%	2.1%	-
950	83.9%	6.7%	9.4%	74.9%	15.3%	8.9%	0.9%	-
1000	84.7%	6.3%	9.0%	76.2%	15.4%	8.5%	-	-
1050	85.6%	5.8%	8.6%	77.1%	14.9%	8.0%	-	-
1100	86.6%	5.3%	8.1%	78.1%	14.5%	7.4%	-	-
1150	87.9%	4.7%	7.5%	79.2%	14.0%	6.8%	-	-
1200	89.3%	3.9%	6.8%	80.5%	13.3%	6.1%	-	-

**Table 4:** Valve seat wear measurements.

Point ID	Valve Seat No. 1			Valve Seat no. 2		
	Before Trail	After Trail	Δ	Before Trail	After Trail	Δ
A	3.21	3.24	0.03	3.11	3.06	−0.05
B	3.20	3.23	0.03	3.12	3.04	−0.08
C	3.26	3.25	0.01	3.03	2.95	−0.08
D	3.38	3.30	−0.08	2.93	2.92	−0.01
E	3.45	3.37	−0.08	2.85	2.88	0.03
F	3.45	3.38	−0.07	2.83	2.86	0.03
G	3.38	3.30	−0.08	2.93	2.94	0.01
H	3.28	3.21	−0.07	3.04	3.01	−0.03
	**Valve seat no. 3**			**Valve seat no. 4**		
	**Before trail**	**After trail**	**Δ**	**Before trail**	**After trail**	**Δ**
A	3.39	3.21	−0.18	3.22	3.03	−0.19
B	3.38	3.20	−0.18	3.18	3.00	−0.18
C	3.45	3.25	−0.20	3.12	2.88	−0.24
D	3.53	3.31	−0.22	2.98	2.82	−0.16
E	3.60	3.35	−0.25	2.94	2.80	−0.14
F	3.63	3.32	−0.31	2.96	2.88	−0.08
G	3.57	3.27	−0.30	3.03	2.96	−0.07
H	3.46	3.18	−0.28	3.18	3.04	−0.14

## Data Availability

Data sharing is not applicable for this article.
